# Properties of A Model Self-Healing Microcapsule-Based Dental Composite Reinforced with Silica Nanoparticles

**DOI:** 10.3390/jfb13010019

**Published:** 2022-02-14

**Authors:** Khaled Abid Althaqafi, Abdulrahman Alshabib, Julian Satterthwaite, Nikolaos Silikas

**Affiliations:** 1 Faculty of Dentistry, College of Dental Medicine, University of Umm Al Qura, Makkah 24211, Saudi Arabia; 2 Division of Dentistry, School of Medical Sciences, University of Manchester, Oxford Road, Manchester M13 9PL, UK; alshabib@yahoo.com; 3 Department of Restorative Dental Sciences, College of Dentistry, King Saud University, Riyadh 11545, Saudi Arabia; 4 Engr. Abdullah Bugshan Research Chair for Dental and Oral Rehabilitation, King Saud University, Riyadh 11545, Saudi Arabia

**Keywords:** self healing, self sealing, microcapsules, resin composites, dental composites

## Abstract

Aim: The purpose of this study was to evaluate the mechanical properties of an experimental self-healing dental composite model (SHDC) composed of SiO_2_ nanoparticles with varying percentages of triethylene glycol dimethacrylate (TEGDMA) monomer and *N*,*N*-dihydroxyethyl-*p*-toluidine (DHEPT) amine microcapsules. Materials and methods: Microcapsules were prepared by in-situ polymerisation of PUF shells, as explained in our previous work. The model SHDC included bisphenol A glycidyl dimethacrylate (Bis-GMA:TEGDMA) (1:1), 1 wt% phenyl bis(2,4,6-trimethylbenzoyl) phosphine oxide (BAPO), 0.5 wt% benzoyl peroxide (BPO) catalyst, 20 wt% silanised silica dioxide (SiO_2_) (15 nm) and (0, 2.5, 5, 7.5, 10 wt%) of microcapsules (120 ± 45 μm). Light transmission, hardness, degree of conversion (DC), flexural strength and elastic modulus of the SHDC model were measured. Results: The degree of conversion of the SHDC ranged from 73 to 76% 24 h after polymerisation. Hardness measurements ranged from 22 to 26 VHN (*p* > 0.05); however, the flexural strength was adversely affected from 80 to 55 MPa with increasing microcapsules of up to 10 wt% in the composites (*p* < 0.05). Conclusion: Only flexural strength decreased drastically ~30% with increasing microcapsules (>10 wt%) in the composites. All other measured properties were not significantly affected. Accordingly, we recommend a stronger composite material that could be created by increasing the filler content distribution in order to achieve a hybrid self-healing composite with enhanced mechanical properties.

## 1. Introduction

Dental composites are commonly used as the restorative material of choice in contemporary dentistry [1,2]. Composites can bond to the tooth structure via bonding agents to repair destructed or decayed teeth with acceptable aesthetics, satisfactory mechanical properties and ease of use [3]. Although substantial efforts have been made to improve the clinical performance and properties of resin composites [4,5,6,7], composite restorations have been shown to encounter two main downsides: secondary caries and bulk fracture [8]. Survival rate for composites has been reported to be 91.7% at 5 years and 82.2% at 10 years [9,10,11]. In many cases, composite failure can be attributed to the accumulation of microcracks, which are generated from masticatory forces and thermal stresses [12].

The introduction of self-healing composites and polymers involving microcapsule systems has shown that providing long-life structural materials is possible [13]. Self-healing composites can be understood as materials that have the capability to recover when mechanical damage occurs [13,14]. The potential for crack repair and recovery of mechanical properties has been achieved in bulk thermosetting polymers [14,15,16,17,18], self-healing fibre-reinforced composites [19,20,21,22,23], self-healing dental composites [24,25,26,27], self-healing adhesives [28], self-healing bonding resins [29], elastomers [30,31] and coatings [32]. Self-healing dental composites are promising materials that could help to produce a stable interface that resists secondary caries and reduces marginal gaps [33]. In addition, self-healing microcapsule-based composites seem to have increased material durability by the recovery of their mechanical properties in the case of cracking and fracturing. This is achieved through the microcapsules rupturing and releasing polymerisable healing agents that are able to seal a crack and stop it propagating [13,19,26].

An initiator is an essential component in the resin composite mixture. In order to facilitate chemical polymerisation with the healing agent involved in the microcapsules [25,26,27], a benzoyl peroxide (BPO) catalyst and an *N,N*-dihydroxyethyl-*p*-toluidine (DHEPT) amine have commonly been used to chemically cure composites [34]. Studies have reported the use of poly(urea-formaldehyde) microcapsules, which encapsulate TEGDMA monomer and DHEPT amine accelerator, as healing agents in self-healing dental composites [25,26,27].

The reinforcing phase consists of dispersed filler particles; it is known that increasing the volume fraction of filler in composites, in general, increases the mechanical properties of the material [35]. The use of silica nanoparticles as a filler in resinous materials has increased in the last 10 years. With the addition of SiO_2_ fillers in composites, an enhancement of properties has been achieved, i.e., an increase in strength, stiffness, toughness, modulus, scratch resistance and fatigue performance [36]. The suggested optimum filler content varies in the current literature and it is highly dependent on the function of the resinous matrix system of choice [36]. However, the use of a higher percentage of SiO_2_ fillers negatively affects the viscosity of the resin and limits its use in certain applications, in addition to promoting the deterioration of the mechanical properties at higher filler volume fractions [36].

In the present study, the focus was to formulate a self-healing dental composite (SHDC) with TEGDMA-DHEPT microcapsules and silica dioxide (SiO_2_) filler nanoparticles. The null hypotheses were as follows: (1) light transmittance and a degree of conversion that was not affected by the addition of TEGDMA-DHEPT microcapsules in the composite; (2) hardness, flexural strength and elastic modulus that was not affected with the addition TEGDMA-DHEPT microcapsules in the composite.

## 2. Materials and Methods

### 2.1. Raw Materials

A model dental composite (SHDC) was formulated from bisphenol A glycidyl dimethacrylate (Bis-GMA) and triethylene glycol dimethacrylate (TEGDMA), phenyl bis(2,4,6-trimethylbenzoyl) phosphine oxide (BAPO) photo-initiator, benzoyl peroxide (BPO) chemical initiator (Sigma–Aldrich Company Ltd., Dorset, UK), silicon dioxide (SiO_2_) silanised with KH220—Dimethoxydiphenylsilane coupling agent (MK nano, MK Impex Corp., Ontario, Canada).

Poly(urea-formaldehyde) microcapsules were formulated from triethylene glycol dimethacrylate (TEGDMA) monomer and *N*,*N*-dihydroxyethyl-*p*-toluidine (DHEPT) amine (Esschem Europe Ltd., Seaham, UK). All chemicals were analytical grade and used as received with no further purification.

### 2.2. Preparation of Self-Healing Dental Composite (SHDC)

The organic phase consisted of Bis-GMA:TEGDMA mixed at a (1:1, *w*/*w*) ratio. BAPO photo initiator was added at 1 wt% and self-healing initiator BPO was also added at 0.5 wt% to the mixture. The inorganic phase consisted of SiO_2_ nanoparticles that were coated with silane coupling agent and dispersed at 20 wt%, with an average particle size of 15 nm. Microcapsules were dispersed gently into the experimental photo-activated composites with low-speed mechanical mixing at the following percentages: 0, 2.5, 5, 7.5 and 10 wt% (Table 1).

The microcapsules were prepared by in-situ polymerisation of poly(urea-formaldehyde) shells encapsulating TEGDMA monomer and 1 wt% DHEPT amine as healing agents. The average microcapsule diameter was between 150 and 300 μm.

### 2.3. Light Transmittance Measurements

Five cylindrical specimens were prepared in each experimental composite group using a PTFE disc mould (4 mm internal diameter, 2 mm height). The mould was placed on a glass slab (2 mm thick) with Mylar strips (Moyco Union Broach, York, ME, USA) on the bottom and top surfaces of the mould and pressed with another glass slab on the top to remove excess resin. One increment was placed for each specimen during preparation.

The prepared SHDC composites were first placed and cured for 20 s in a MARC^TM^ spectrophotometer resin calibrator (BlueLight Analytics Inc., Halifax, NS, Canada). The light curing unit used was a 455 (±10) nm LED light (S10 Elipar, 3M ESPE, St Paul, MN, USA). Prior to experimentation, the maximum light irradiance of the top sensor of the resin calibrator was recorded, and the mean irradiance over 20 s duration was reported. After curing, specimens were demoulded and incubated for 24 h at 37 °C prior to hardness evaluation.

### 2.4. Hardness Measurements

A hardness instrument (FM-700, Future-Tech Corp., Kawasaki, Kanagawa, Japan) was used to measure the top and bottom surfaces for the formulated composites. A Vickers indenter was used with an applied load of 300 gf (2.94 N) for 15 s. Five indentations were measured for top and bottom surfaces, with a distance of four times the diagonal length of the indenter between each indentation (4D, around 200 nm). In order to have standardised readings, indentations were made randomly in the matrix or microcapsules surfaces. The mean of five readings for each specimen was recorded. In addition, the means and the standard deviations of the five specimens in each group were calculated and recorded.

### 2.5. Degree of Conversion Measurements

A Fourier transfer-infrared spectrometer (FT-IR) was used to measure the unpolymerised carbon–carbon double bond (Avatar 360, Nicolete Analytical Instrument, Thermo Electron Corp., Cambridge, UK). The spectra of five specimens in each group were recorded. A mould (PTFE disc, 4 mm internal diameter, 0.5 mm height) was placed on top of the diamond ATR crystal reader in the FT-IR, filled with resin composite paste, covered on the top surface with a Mylar strip and pressed to remove the excess resin. It was then photo cured for 40 s using a 455 (±10) nm LED light.

The DC was reported using the spectra of the composite immediately post-cure and 24 h after polymerisation. Specimens were stored in distilled water and incubated at 37 °C for 24 h. The means and standard deviations of the post-polymerisation spectra were recorded.

The spectra of the composites were recorded over the range of 4000 to 400 cm^−1^, with 32 scans at a resolution of 4 cm^−1^. Software was used to record the peak intensity ratio of aliphatic C=C at 1637 cm^−1^ against the internal reference immediately post-cure and 24 h after polymerisation. Meanwhile, the aromatic C=C peak at 1608 cm^−1^ was selected as the internal reference.

The following equation was used to calculate the degree of conversion (Equation (1)):

(1) $$DC\;peak\;\%=\left[1-\frac{Polymer\left(C=C/C=O\right)peak\;height\;after\;curing}{Monomer(C=C/C=O)peak\;height\;before\;curing\;}\right]\times 100$$

Equation (1): degree of conversion from peaks on the FT-IR spectrum (C = C_aliphatic_ 1637 cm^−1^, C = C_aromatic_ 1608 cm^−1^).

### 2.6. Flexural Strength Measurements

The prepared SHDC composite paste was placed in a PTFE mould (2 mm × 2 mm × 25 mm) on a glass slab (2 mm thick) separated with Mylar strips on the bottom and top surfaces of the mould, then pressed with another glass slab on the top to remove the excess resin. Each resin specimen was photo cured in five overlapping points for 20 s on each open side of the mould using a 455 (±10) nm LED light. Upon light curing from one point to another on the specimen, the curing tip was positioned on half the diameter of the cured area by the earlier exposure to ensure optimal polymerisation of the entire specimen. After curing, specimens were demoulded and sharp edges were smoothed with P800 grit SiC abrasive paper. All specimens were stored in distilled water at 37 °C for 24 h prior to testing.

A computer-controlled universal testing machine (Zwick/Roell Z020, Ulm-Einsingen, Germany) was used to measure the flexural strength of the specimens in a three-point flexure bending test that used a span of 20 mm, a crosshead speed of 1 mm/min and a load cell of 500 N. Prior to testing, five specimens in each group were removed from the storage medium (distilled water). The thickness of each composite specimen was measured at the centre and the two ends of the specimen using a digital caliper (Mitutoyo, Tokyo, Japan), and the mean thickness was used for cross sectional area estimations.

The following equation was used to calculate the flexural strength (MPa) (Equation (2)):

(2) $$S=3P_{max}\;L∕\left(2bh^{2}\right)$$

Equation (2): *S*—flexural strength; *P_max_*—the load at failure; *L*—span; *bh*—specimen width and thickness.

The elastic modulus was determined from the slope of the linear region of the load deflection curve, and the following formula was used to calculate the elastic modulus (GPa) (Equation (3)):

(3) $$E=\left(P/d\right)\;\left(L^{3}/\left[4bh^{3}\right]\right)$$

Equation (3): *E*—elastic modulus; *P*—load; *d*—displacement of the slope in the linear elastic region of the load displacement curve.

The fractured surfaces of selected composite specimens, which were prepared by sputter coating with gold (7 nm), were examined by SEM (Zeiss EVO60, Oberkochen, Germany), to investigate the fracture mechanism of the microcapsules in the resin composite at the fracture plane.

### 2.7. Statistical Analysis

Collected data were statistically analysed using SPSS software (IBM SPSS Statistics 23.0, SPSS Inc., Chicago, IL, USA). The Shapiro–Wilk test was implemented to confirm the normality of the data. Levene’s test also confirmed the equality of variance.

One-way analysis of variance (ANOVA) and independent sample *t*-tests were conducted to detect any significant effects of variables for the study groups at a significance level of (*p* ≤ 0.05). All data were plotted using Sigma Plot (SigmaPlot 13.0, Systat Software Inc., San Jose, CA, USA).

## 3. Results

### 3.1. Light Transmittance Measurements

**Figure 1:**
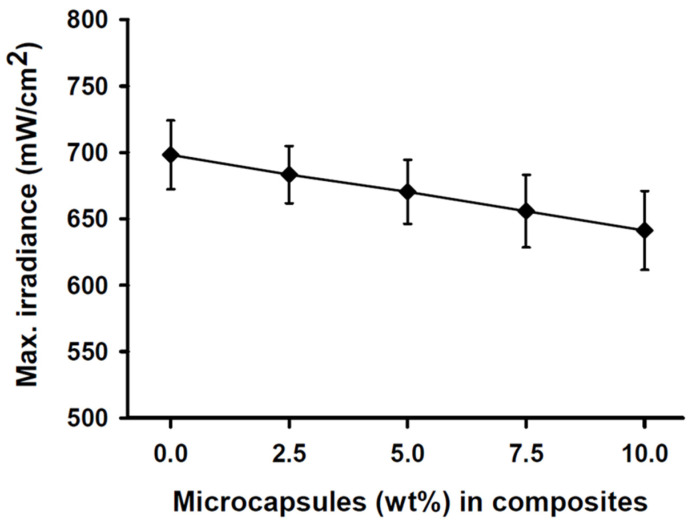
Maximum irradiance measurements of the SHDC groups. The transmittance through the 2 mm-thick resin was recorded at 698 (±26) mW/cm^2^ with the MC_0_ control group steadily decreased to reach 641 (±42) mW/cm^2^ at the MC_10_ group (mean ± sd; *n* = 5). Diagram is reused from [37].

The maximum light irradiance measurement of the light curing unit at the top sensor of the resin calibrator was 2050 (±24) mW/cm^2^ over 20 s prior to experimentation. The light transmittance through the SHDC did not show any statistical differences between any of the groups (*p* > 0.05). The light transmittance through the 2 mm-thick specimens was found in the control group’s MC_0_ composite at 698 (±26) mW/cm^2^, while the MC_10_ composite showed a lower value of 641 (±42) mW/cm^2^ (Figure 1).

### 3.2. Degree of Conversion Measurements

The mean degree of conversion values immediately after curing the (DC_0_) of MC_2.5_ to MC_10_ microcapsules in the composite was around 67%, with no statistical differences between test groups (*p* > 0.05). The DC_0_ value of 68% was shown in the MC_5_ group. However, the control group, MC_0_, showed a value of 65% at DC_0_, which was statistically different compared to MC_2.5_ and MC_5_ (*p* < 0.05). The mean degree of conversion 24 h after polymerisation (DC_24_) was between 73 and 76%, with the highest score being for the control group, MC_0_. The DC_24_ values showed no statistical differences between any of the groups (*p* > 0.05).

**Figure 2:**
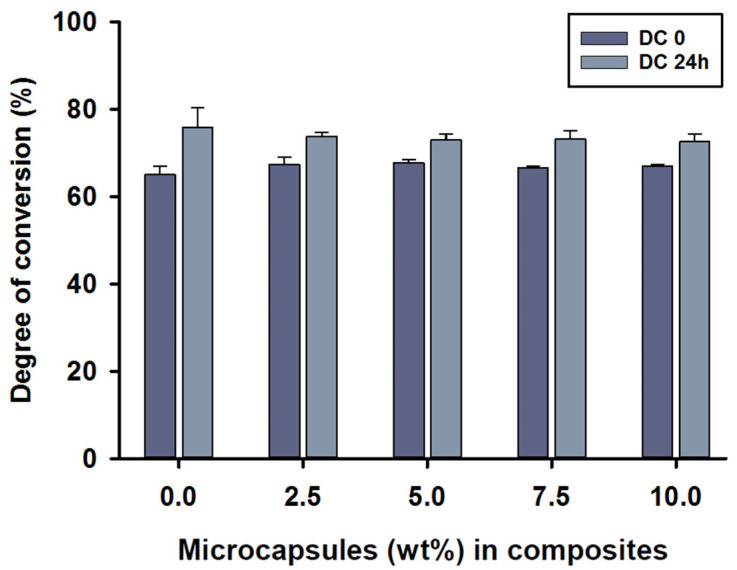
Degree of conversion (%) values of the SHDC, including DC_0_ and DC_24_ (mean ± sd; *n* = 5). None of the DC values showed any statistical differences between the groups with different microcapsule percentages (*p* > 0.05). Some differences could be noticed in DC_0_ between the control group, MC_0_, and both MC_2.5_ and MC_5_ groups (*p* < 0.05). Differences in values between DC_0_ and DC_24_ were shown in MC_0_, MC_7.5_ and MC_10_ (*p* < 0.05). Diagram is reused from [37].

Intragroup comparisons were also performed by independent sample *t*-tests between DC_0_ and DC_24_. There were statistically significant differences in all composite groups between the DC_0_ and DC_24_ measurements (*p* < 0.05) (Figure 2).

### 3.3. Hardness Measurements

**Figure 3:**
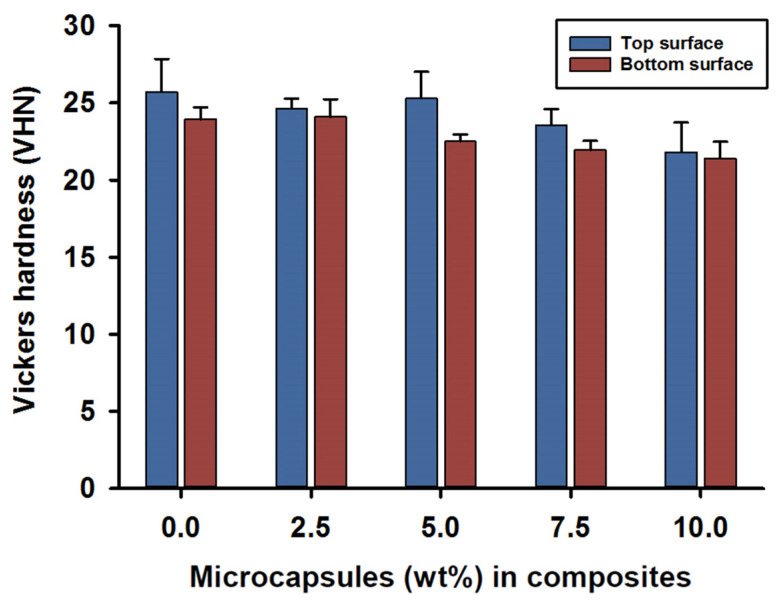
Vickers hardness (VHN) of the SHDC groups. Top and bottom surface values recorded as (mean ± sd; *n* = 5). The bar chart shows higher scores in the top surface hardness measurements of the SHDC, with an obvious decrease in the values with increasing microcapsules percentages in the composite mixture. The top surface measurements show statistical differences in MC_10_ compared to MC_0_ and MC_5_ (*p* < 0.05). The bottom surface measurements show statistical differences in MC_0_ and MC_2.5_ compared to MC_7.5_ and MC_10_ (*p* < 0.05). Statistical differences in values between the top and bottom surfaces were shown in MC_5_ and MC_7.5_ (*p* < 0.05). Diagram is reused from [37].

Vickers hardness (VHN) measurements for the top and bottom surfaces were recorded at 24 h after photo curing for the SHDC groups. The mean value for the top surface hardness was in the control group MC_0_ at 26 (±2) VHN, while lower values were recorded for the MC_7.5_ and MC_10_ groups—around 22 (±2) VHN. There were statistical differences between MC_10_ and both MC_0_ and MC_5_ (*p* < 0.05); however, no significant differences were reported for the rest of the groups (*p* > 0.05) (Figure 3).

The hardness of the bottom surfaces showed similar results compared to the top surfaces; mean values of 23 (±1) VHN were recorded for the MC_0_ and MC_2.5_ groups. However, lower hardness values of 21 (±1) VHN were recorded on the bottom surfaces for composites MC_7.5_ and MC_10_. Statistical differences in the hardness values of the bottom surfaces were found in the MC_0_ and MC_2.5_ groups when compared to the MC_7.5_ and MC_10_ groups (*p* < 0.05); no difference was recorded for the rest of the groups (*p* > 0.05).

Intragroup comparisons between the top and bottom surface hardness values indicated statistical differences in the MC_5_ and MC_7.5_ groups (*p* < 0.05); however, the rest of the groups showed no statistically significant differences between the top and bottom surface values (*p* > 0.05) (Figure 3).

### 3.4. Flexural Strength Measurements

**Figure 4:**
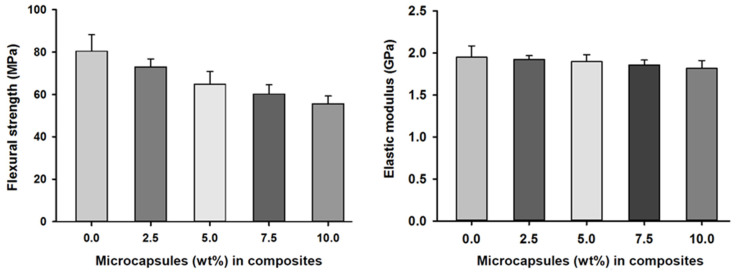
Flexural strength and elastic moduli of the SHDC groups containing different microcapsules (%) (mean ± sd; *n* = 5). The flexural strength of the composite was negatively affected by increasing the percentages of microcapsules in the composite with statistically significant differences between groups MC_0_ and MC_5_ and groups MC_7.5_ and MC_10_ (*p* < 0.05). The elastic modulus of the SHDC did not show any statistical differences between any of the groups (*p* > 0.05). Diagram is reused from [37].

Flexural strength and elastic modulus measurements of the SHDC groups showed values of 80 (±12) MPa and 1.95 GPa in the control group, MC_0_. Reduced values of 55 (±4) MPa and 1.81 GPa were found in the MC_10_ group. There were statistically significant differences between the control group, MC_0_, and the MC_5_, MC_7.5_ and MC_10_ groups; similar differences were also noticed in MC_2.5_ compared to MC_7.5_ and MC_10_ (*p* < 0.05). The elastic modulus measurements of the SHDC groups showed no statistically significant differences between the groups (*p* > 0.05) (Figure 4).

**Figure 5:**
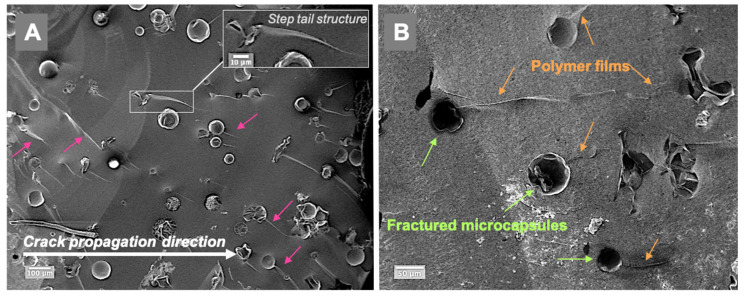
SEM images of the fractured composite surfaces in a composite model consisting of 5 wt% microcapsules (Zeiss, EVO60, high vac, 5.00 kV, Mag (**A**) 200×, (**B**) 500×). (**A**)—a step nail structure following the crack propagation direction is demonstrated (arrows), and a higher magnification is presented in the inset A. (**B**)—the presence of released and polymerised films from the cracked and ruptured microcapsules in the microcracks, as shown in the fractured self-healing composite surface. Diagram is reused from [37].

Representative fractured surfaces of self-healing composites are illustrated in Figure 5; the examined specimens contained 5 wt% microcapsules. The fractured surfaces showed typical step-nail patterns in the same direction as the crack propagation (Figure 5A). Polymer film formation at the microcracks in the composite can be seen in Figure 5B. The cracked microcapsules released TEGDMA-DHEPT healing agent then polymerised. Size variation of microcapsules can be shown in Supplementary Materials Figure S1.

## 4. Discussion

In our previous work, TEGDMA-DHEPT microcapsules were successfully synthesised via emulsion polymerisation to achieve poly(urea-formaldehyde) capsular shells [38,39]. Microcapsules were prepared by in-situ polymerization where EMA acted as a surfactant, which helped to form an oil-in-water (O/W) emulsion (the oil being TEGDMA-DHEPT liquid). Our findings from the SEM imaging showed that PUF nanoparticles existed on the external surface of the capsular shells. PUF nanoparticles in the outer surface of the microcapsules can facilitate the mechanical interlocking interface (micromechanical retention) to the host resin polymer matrix upon photo activation (light curing) [26]. This retention interface allows the microcapsules to break when subjected to cracking. However, if the outer surface of the microcapsules had a smooth, non-porous morphology, the interlocking interface would be missing and the crack could bypass the microcapsules without breaking them, resulting in no healing liquid to fill the crack [26].

Regarding the biocompatibility of the self-healing system, which includes TEGDMA monomer and DHEPT amine in the microcapsules and BPO catalyst within the resin composite matrix, the materials were all approved by the Food and Drug Administration, and are available in commercial resin-based dental composites. In addition, human gingival fibroblast cytotoxicity tests in vitro have proven that TEGDMA-DHEPT microcapsules exhibit a good biocompatibility, hence the incorporation of microcapsules in resin did not drastically compromise the cell viability [26]. However, the possibility of leakage of unreacted free formaldehyde from PUF shells should be investigated.

Self-healing dental composites (SHDCs) were formulated by incorporation of Bis-GMA: TEGDMA (1:1), 20 wt% SiO_2_ nanoparticles (15 nm) and different percentages of TEGDMA-DHEPT microcapsules (0, 2.5, 5, 7.5, 10 wt%) with an average diameter of 120 ± 45 μm (*n*: 100). The photo-activated resin composite had 1 wt% of type I photo initiator phenyl bis(2,4,6-trimethylbenzoyl) phosphine oxide (BAPO), which generated free radicals by fragmentation of the photo-initiator molecule. This is unlike type II photo-initiator systems, which need a co-initiator to donate electrons and protons in their excited state to generate the radicals for polymerisation [40]. A 0.5 wt% BPO initiator was also added to promote chemical or auto-polymerisation by free radical formation, which in turn targeted the carbon double bond (C=C) leading to polymerisation [34]. The most commonly used activator is tertiary aromatic amine *N,N*-bis(2-hydroxyethyl)-*p*-toluidine, which reacts with the initiator BPO [34].

During photo polymerisation, light transmitted through a resin composite is absorbed and scattered; this causes the light intensity to be attenuated and its efficiency drops as the depth increases [41,42]. Many factors affect the depth of cure, such as light irradiance [43], exposure time [44,45], materials composition [46], resin composite shades [47] and translucency [48,49]. Light scattering is the most important limiting factor for the depth of cure and it is maximised when the filler particle size is near to half of the wavelength emitted by the light source [50]. Studies have shown that other factors can also influence the light transmittance of resin-based composites, such as fillers and the polymeric matrix refractive index, filler type and content and monomer type [51,52]. The light transmittance through the SHDC in the present study showed no statistically significant differences between the groups (*p* > 0.05). The differences in the maximum light transmittance measurements between the experimental groups were mainly due to microcapsules’ particle sizes of ≥150 μm.

The preferred mode of cure in resin-based dental composites is photo polymerisation. Ideally, the photo-polymerisation of the restorative dental composite will convert all monomers to polymers [53]; yet, most of dimethacrylate monomers showed a degree of conversion (DC) ranging from 55 to 85% under conventional curing conditions [54,55,56,57] and a further reduction in DC rate could be associated with depth [53]. The mechanical properties of composites are directly influenced by the degree of conversion [58].

The degree of conversion of the SHDC in this study ranged from 65 to 68% immediately after polymerisation (DC_0_). After 24 h at 37 °C in distilled water (DC_24_), scores higher than DC_0_ were noted, ranging from 73 to 76%. The degree of conversion increased after 24 h in conditions matching the intraoral temperatures and hydrophilic environment of the oral cavity. A recent study reported that a higher degree of conversion could be seen after 24 h [59]. The type and concentration of photo initiator can influence the degree of conversion in the resin matrix. The high reactivity of the BAPO photo initiator seems to improve the mechanism of free radical formation. As absorption of light energy (BAPO*) takes place, the molecule undergoes α-cleavage in the excited triplet state of the C–P bond; this may occur twice, which results in four free radicals being generated per molecule [60]. In other words, the first free radical is less reactive than the second radical by 2 to 6 times [61].

BAPO photo initiators, according to UV–VIS spectra, have a λ_max_ value ranging from 365 to 440 nm. The λ_max_ of BAPO was greater than 400 nm [62]. BAPO has proven advantages, such as a white colour that helps light penetrate and increases the polymerisation depth in the resin [63,64]. Although the most commonly used photo-initiator system in dental resin composites is type II (e.g., camphorquinone, CQ), this requires an amine co-initiator (e.g., DMAEMA) to generate free radical polymerisation [65]. This may compromise the aesthetics of the dental restoration as the CQ molecule is yellow and the amine undergoes yellowing over time [65,66,67]. The model self-healing composite requires the addition of a BPO initiator in the resin matrix, which requires the use of an amine co-initiator with a CQ photo initiator to prevent resin matrix polymerisation with BPO upon mixing [68].

Surface hardness of self-healing composites has not been widely explored in the literature. The presented study reported Vickers hardness (VHN) for the SHDC, however, the VHN scores were considered low (22–26 VHN) when compared to commercial dental composites. The surface hardness of dental composites is affected by the volume fraction of fillers rather than the filler particle hardness [69]. Composite hardness is also affected by hydrolytic degradation and water sorption [70,71]. Material hardness is strongly associated with compressive strength, degree of conversion and wear resistance [72,73]; low hardness values may indicate a poor chemical/physical bonding in the matrix/filler interface [73].

The flexural strength and elastic modulus of SHDCs can provide insight into the effect of microcapsules in dental composites on the mechanical properties of the material. According to ISO standard 4049:2009 of polymer based restorative materials, flexural strength values shall be equal to or greater than 80–100 MPa. However, the flexural strength values of the experimental SHDCs ranged from 55–80 MPa (MC_10_ to MC_0_ groups, respectively). Flexural strength values significantly decreased with the addition of 7.5 to 10 wt% microcapsules into the composite. The elastic moduli of the SHDCs did not show significant differences between the groups (1.95—1.81 GPa from MC_0_ to MC_10_). These findings were in agreement with another study involving TEGDMA-DHEPT microcapsules in dental composites, which concluded that at 15 wt% of microcapsules in the composite the flexural strength dropped drastically, and at 20 wt% of microcapsules the value reached 30 MPa, indicating deterioration of the mechanical properties [26]. The inclusion of microcapsules into resin composites may have an adverse effect in the mechanical properties of the composite. This is due to the size and strength of microcapsules’ shells, as they hold the healing liquid. Thus, this property of the microcapsule is reduced and deteriorates the mechanical properties of the composites.

The mechanical properties of fibre-reinforced composites (FRCs) differ considerably from conventional and bulk particulate-filled composites (PFC), as they display superior fracture toughness [74,75]. One approach aimed at improving the mechanical properties of SHDCs is to use millimetre-scale, discontinuous fibre-reinforced composites (millimetre-scale, discontinuous-FRCs). Fibre toughening mechanisms rely on their ability to deflect crack propagation, to stretch, bridge and resist the opening and further spreading of the crack. These properties induce a closure force onto the crack itself [76]. This action subsequently lowers the stress concentration at the tip of the crack, thus slowing or preventing further progression. Further study is warranted to investigate the suitability of using fibres in SHDCs.

SEM of the fractured surfaces of the self-healing composite specimen indicated possible polymer film formation into the microcracks at the fracture plane. This indicated that in the presence of a BPO initiator in resin composites, and upon microcapsule rupture, the healing agent is released. The amine accelerator, DHEPT, in the healing agent had the ability to polymerise once it contacted the BPO, then monomer polymerisation sealed the microcracks. The polymerisation capability of the healing agent (TEGDMA-DHEPT) and BPO initiator was also consistent with FT-IR spectra results; a degree of conversion of 60.3% was showed in our previous report.

The incorporation of microcapsules in resin composites can change the properties of the host polymeric material; the goal was to obtain a self-healing capability without diminishing the original mechanical properties of the material. The durability and reliability of the restorative material could be compromised by weaker mechanical properties, including flexural strength and fracture toughness [77,78,79]. Previous reports on the incorporation of microcapsules in resin-based materials showed significant improvements in tensile strength [80] and flexural strength in the polymer matrix [81]. Yet, other reports showed a continuous reduction in the strength of the resin polymer with high percentages of microcapsule involvement [82].

## 5. Conclusions

TEGDMA-DHEPT microcapsules were synthesised by in-situ polymerisation of an O/W emulsion. The microcapsules had the ability to polymerise when they were ruptured and triggered by a BPO catalyst in the host composite. The self-healing dental composite (SHDC) incorporated Bis-GMA:TEGDMA (1:1), 1 wt% BAPO photo-initiator, 0.5 wt% BPO catalyst, 20 wt% silica dioxide nanoparticles and different percentages of microcapsules. The composite properties were not drastically affected in terms of light transmittance, degrees of conversion, hardness and elastic moduli. Microcapsule inclusion did not improve the mechanical properties; rather, a reduction of ~65% in flexural strength measurements showed at 10 wt% of microcapsules in the composite. A way to improve the properties of SHDCs is to increase the filler content percentage with different filler size distributions in order to have a hybrid composite incorporating nano-, micro- and macro-sized particles. The current study suggested that improvements in mechanical properties of SHDCs are required in order to meet the clinical requirements of a restorative material.

## Figures and Tables

**Table 1:** Microcapsules and SiO_2_ wt% in the self-healing dental composites.

Groups	Microcapsules (wt%)	SiO_2_ (wt%)	Total Fillers (wt%)
MC_0_ (control)	0.0	20	20
MC_2.5_	2.5	20	22.5
MC_5_	5.0	20	25
MC_7.5_	7.5	20	27.5
MC_10_	10	20	30

## Data Availability

Not applicable.

## Supplementary Materials

The following supporting information can be downloaded at: https://www.mdpi.com/article/10.3390/jfb13010019/s1, Figure S1: Size variation of microcapsules.
