# Supplementary figures and images for: Properties of A Model Self-Healing Microcapsule-Based Dental Composite Reinforced with Silica Nanoparticles
Source: J Funct Biomater. 2022 Feb 14;13(1):19. doi: 10.3390/jfb13010019 (PMC8883938; doi:10.3390/jfb13010019)

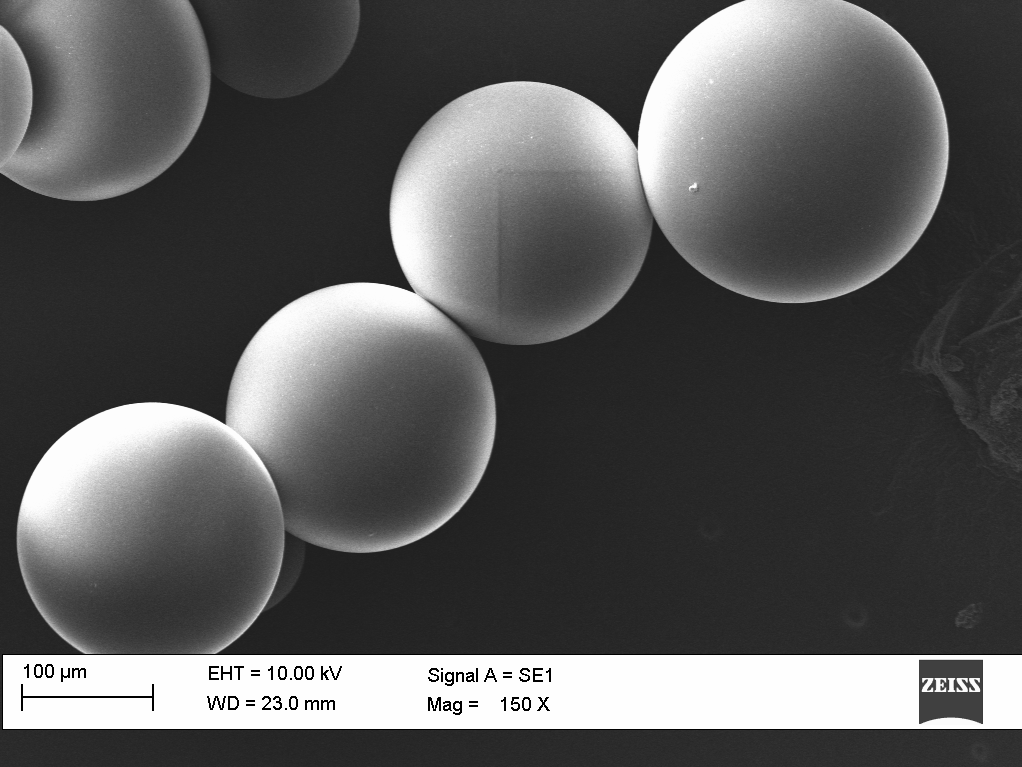

Supplement: Supplementary file 1 [file jfb-13-00019-s001.zip › Figure S1.tiff]
